# Development and Validation of the Professional Self‐Concept Scale for Hospital Nurses (PSCS‐HN)

**DOI:** 10.1002/nur.70059

**Published:** 2026-02-14

**Authors:** Eun‐Ha Kim, Hye‐Ah Yeom

**Affiliations:** ^1^ College of Nursing The Catholic University of Korea Seoul South Korea

**Keywords:** factor analysis, statistical, Nursing staff, hospital, self‐concept, surveys and questionnaires

## Abstract

Professional self‐concept (PSC) has a positive influence on the attitudes and behaviors of members of an organization toward their work. Although establishing PSC is vital for nurses to provide quality nursing care, assessing PSC precisely in ever‐changing healthcare environments can be challenging. This methodological study involved the development of a new instrument, the Professional Self‐Concept Scale for Hospital Nurses (PSCS‐HN) and evaluation of its validity and reliability. The scale development phase involved the development of preliminary items based on a literature review and focus group interviews, followed by primary and secondary content validation by an expert panel. In the scale evaluation phase, a survey of 440 nurses working in general hospitals was conducted to examine scale validity and reliability. Construct validity was evaluated through item, exploratory factor, and confirmatory factor analyses, which resulted in a final scale with five factors and 23 items. Concurrent and convergent validity of the PSCS‐HN were confirmed by significant correlation coefficients of the scale with existing instruments. Regarding reliability, in this study, the Cronbach's alpha and McDonald's ω of the PSCS‐HN were 0.93 and 0.95, respectively. The results confirm that the PSCS‐HN is a valid and reliable scale to assess hospital nurses' PSC.

## 1 Introduction

Professional self‐concept (PSC) refers to professionals' beliefs and attitudes about themselves, formed through self‐evaluation of their expertise, values, and skills (Arthur 1992). For a nurse, PSC refers to a conscious view of oneself as a nurse, and marks the beginning of a conscious effort to grow as a nursing professional (Weis and Schank 2002). Nurses who have established a positive PSC can deal with conflicts that occur in clinical settings and are thus capable of solving problems and efficiently performing their professional duties (Park et al. 2016).

Hospital nurses' PSC has been known to affect their positive career outcomes. PSC is essential for nurses to achieve academic growth as professionals (Arthur 1995), and successfully perform their professional roles (Arthur 1990). PSC positively influences the behaviors and attitudes of members of an organization toward their work.

While nurses with a positive PSC are more satisfied with their jobs and more engaged in their organizations, those with a low PSC tend to frequently experience feelings of tension and fatigue, lose motivation due to decreased performance, and become less communicative with patients and colleagues, all of which negatively impact their ability to perform their nursing duties effectively (Maslach 2003). Nurses whose careers align with their self‐concept perceive their careers as meaningful and rewarding, which is instrumental in their providing quality patient care. Conversely, nurses with a negative self‐concept may feel disappointed in their abilities, lack motivation for their work, and experience decreased job satisfaction (Miao et al. 2024). Thus, PSC is important for nurses to develop a professional perspective and efficiently perform nursing duties in harmony with others in a medical setting where diverse professions work together.

The most commonly used instruments to measure nurses' PSC are the Professional Self‐Concept of Nurses Instrument (PSCNI) (Arthur 1995), Nurses Self‐Concept Questionnaire (NSCQ) (Cowin 2001), and Nursing Self‐Concept Instrument (NSCI) (Angel et al. 2012). Table 1 presents an outline of these scales. PSCNI has been widely used to measure nurses' PSC. However, the scale was developed three decades ago, and recent changes in healthcare environments, such as an aging nursing workforce and nurses' perceived roles in clinical practice are not reflected, thus warranting updates to the existing scale. The NSCQ is a lengthy instrument comprising 36 items and has limitations in clearly distinguishing the difference between communication with others and staff relation factors, thereby emphasizing the need for a more concise instrument. The NSCI was primarily developed for nursing students, and components of organizational efficiency such as burnout and organizational commitment are not fully reflected in the scale. According to the COnsensus‐based Standards for the selection of health Measurement INstruments (COSMIN) criteria, the PSCNI demonstrated insufficient internal consistency and was rated as not recommended (C). Additionally, methodological limitations in content validity and structural validity were identified during the translation and adaptation processes. Meanwhile, possible shortcomings of the NSCI and NSCQ include inadequate involvement of target populations in content validity assessment and insufficient evaluation of cross‐cultural and construct validity (Zhou et al. 2023). Therefore, although these instruments demonstrate reliability in certain measurement properties, they show some deficiencies in key aspects of validity, limiting their ability to accurately capture PSC. Accordingly, considering the need for developing updated instruments that reflect the cultural and practical backgrounds of nurses in ever‐changing healthcare environments, this study aimed to develop a scale to measure hospital nurses' PSC.

**Table 1:** Literature review of measurement instruments.

Instrument	Author(s)	Year	Number of items	Rating scale	Participants	Dimensions
PSCNI (Professional Self‐Concept of Nurses Instrument)	Arthur	1992	27	Four‐point Likert scale	Nursing students/Registered nurses	1. Professional practice (flexibility, skill, leadership) 2. Satisfaction 3. Communication
	Arthur	1995	27	Four‐point Likert scale	Nursing students	1. Professional practice 2. Satisfaction 3. Communication
NSCQ (Nurses' Self‐Concept Questionnaire)	Cowin	2001	36	Eight‐point Likert scale	Nursing students/Registered nurses	1. General nursing 2. Care 3. Staff relations 4. Communication 5. Knowledge 6. Leadership
NSCI (Nurses' Self‐Concept Instrument)	Angel et al.	2012	14	Eight‐point Likert scale	Nursing students	1. Care 2. Knowledge 3. Staff relations 4. Leadership

## 2 Methods

**Figure 1:**
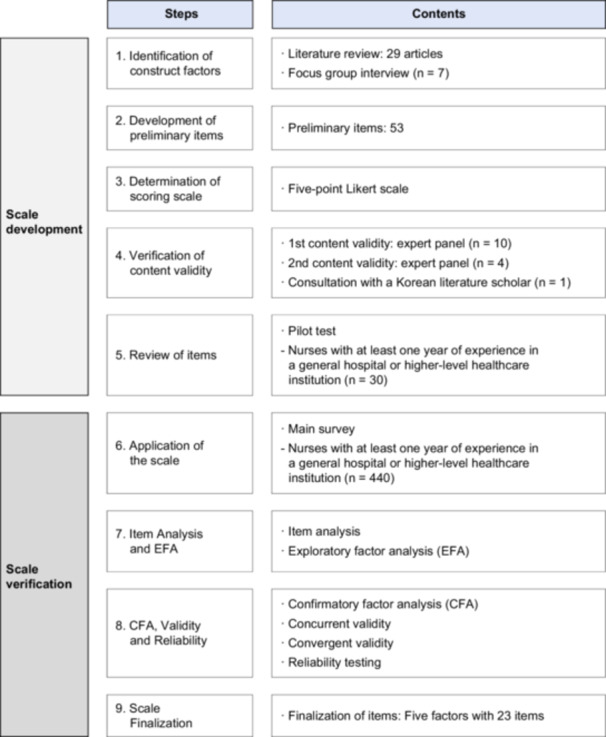
Process of scale development.

This methodological study involved the development of the Professional Self‐Concept Scale for Hospital Nurses (PSCS‐HN) and verification of its reliability and validity. The development and verification process of PSCS‐HN was conducted in nine steps according to DeVellis and Thorpe's (2021) guidelines by dividing them into the scale development and verification stages (Figure 1).

### 2.1 Phase 1. Scale Development

#### 2.1.1 Step 1: Identification of Construct Factors

This study employed the hierarchical model of self‐concept developed by Shavelson et al. (1976) as a conceptual framework. This model is divided into two categories: academic and non‐academic self‐concept. Academic self‐concept refers to an individual's perceptions related to educational achievement, while non‐academic self‐concept constitutes the physical, social, and emotional aspects related to one's self‐concept. Based on this model, we classified construct factors and attributes derived from a literature review and focus group interviews, described further below. A literature review was conducted using combinations of the following keywords: “nurses,” “nurse,” “professional nursing,” “self‐concept,” “professional self,” “self‐concept instrument,” and “PSC.” Six databases—CINAHL, Embase, Google Scholar, PubMed, KERIS, and KISS— were searched regardless of publication period. Studies were included if they targeted nurses and were published in English and Korean. Conference abstracts, articles without full texts, and research posters were excluded. When both a dissertation and journal article were available, only the journal article was included in the review.

A literature search yielded 5892 studies (5485 international and 407 domestic). After removing duplicates, 2941 titles and abstracts were screened, of which 2744 were excluded for irrelevance. The abstracts and full texts of the remaining 197 articles were reviewed, resulting in the inclusion of 29 studies in the final analysis: 23 on hospital nurses' PSC (eight domestic, 15 international) and six on measurement tools (two domestic, four international). Study quality was evaluated based on methodological clarity, study design, and relevance to PSC.

Further, focus group interviews were conducted using a semi‐structured interview guide to explore participants' perceptions regarding PSC and related experiences, influencing factors, and interpretations. Participants were asked to verbalize their understanding and opinions of each item in the guide, and all responses were audio‐recorded, transcribed, and analyzed to derive the relevant attributes.

#### 2.1.2 Step 2: Development of Preliminary Items

The preliminary scale items were composed based on the attributes derived from the literature review and focus group interviews. To reduce ambiguity in item interpretation, each item was developed to measure only a single, independent attribute.

#### 2.1.3 Step 3: Determination of Scoring Scale

Following the development of preliminary items, the scoring system of the scale was determined.

#### 2.1.4 Step 4: Verification of Content Validity

A group of experts was invited for item evaluation to verify the content validity of the derived items. A total of two rounds of content validity verification were conducted. The content validity questionnaire was designed to collect responses on any improvements needed for each item. To mitigate the tendency of respondents to select a neutral midpoint (Polit and Beck 2006), a four‐point scale was employed for the content validity questionnaire. According to established standards, the number of experts for content validation should be no less than three and no more than 10 (Lynn 1986); accordingly, in this study, the first round involved 10 experts (three nursing professors, one nurse with a PhD, and six nurses with at least 7 years of clinical experience). Based on the findings, the second round was conducted with four nurses who were selected from the 10 experts in the first round of validation.

#### 2.1.5 Step 5: Review of Items

A preliminary survey was conducted with 30 nurses to assess the clarity of the wording, meaning, and intent of the items selected through content validation. Inclusion criteria were nurses who had more than 1 year of experience in a general hospital or higher‐level healthcare institution and performed nursing work that involves direct interactions with patients. The survey was conducted online, and the level of understanding of each item was assessed using a four‐point Likert scale.

### 2.2 Phase 2. Scale Verification

#### 2.2.1 Step 6: Application of the Scale

A large sample survey was conducted for scale verification, in which 440 hospital nurses participated. The inclusion criteria were identical to those for the preliminary survey. Those working in departments with limited direct communication with patients were excluded. A structured questionnaire comprising 106 items was used. We set our overall target sample size using a common planning heuristic for factor analysis (≈ 5–10 participants per item) for the initial 47‐item pool. We recruited 458 participants; after excluding 18 insufficient‐effort cases, 440 remained. To avoid inflating construct validity by analyzing the same respondents with both exploratory factor analysis (EFA) and confirmatory factor analysis (CFA) (Hinkin 1998), we randomly split the sample into two independent subsamples (*n* = 220 each) and conducted EFA and CFA separately. The heuristic informed the total target *N*; the split was used solely to ensure independence, which reduces per‐analysis precision and should be considered when interpreting the EFA and CFA results.

#### 2.2.2 Step 7: Item Analysis and EFA

As the theoretical framework of this study was established based on the general population, EFA was performed to determine its applicability within the professional context of nurses. Prior to conducting EFA, to ensure data quality, we flagged outliers using standardized scores (|*z* | > 3.29) and Mahalanobis D² *(p* < 0.001), and assessed the presence of missing data. EFA models with and without outliers were estimated and compared under identical specifications to assess the robustness of the findings.

The construct validity of the scale was evaluated using EFA. Because the responses were Likert‐type, we computed a polychoric correlation matrix and conducted the analysis accordingly (Kiwanuka et al. 2022). Sampling adequacy was assessed with the Kaiser–Meyer–Olkin (KMO) index and Bartlett's test of sphericity using the polychoric correlation matrix. Factors were extracted with principal axis factoring (PAF), and promax rotation was applied. The number of factors was determined by integrating the Kaiser criterion (eigenvalues > 1.0), the scree plot, and parallel analysis. Model adequacy was judged using the following criteria: primary factor loadings ≥ 0.50, communalities ≥ 0.50, and total variance explained ≥ 60% (Kang 2013).

#### 2.2.3 Step 8: CFA, Validity and Reliability

##### 2.2.3.1 CFA

Missing data were examined prior to analysis. Univariate outliers were identified using standardized scores (|*z* | > 3.29), and multivariate outliers were detected using Mahalanobis D² (*p* < 0.001). CFA models with and without outliers were estimated and compared under identical specifications to assess the robustness of the findings.

CFA was conducted to evaluate the appropriateness of the factor structure identified in EFA. Parameter estimates were obtained using the WLSMV estimator based on a polychoric correlation matrix. The χ²/df ratio was calculated to examine the discrepancy between the observed covariance matrix and the model‐implied covariance matrix, with values ≤ 3.0 indicating acceptable fit. Model fit was evaluated according to the following criteria: root mean square error of approximation (RMSEA) ≤ 0.06, standardized root mean square residual (SRMR) ≤ 0.08, goodness‐of‐fit index (GFI) ≥ 0.95, and incremental fit indices (CFI, TLI) ≥ 0.95. Factor loadings, convergent factor validity, and discriminant factor validity were evaluated. Convergent factor validity was assessed by examining construct reliability (CR ≥ 0.70) and average variance extracted (AVE ≥ 0.50) for each factor. Discriminant factor validity was examined using the Fornell–Larcker criterion, whereby the square root of each factor's AVE exceeded its correlations with other factors, and the Heterotrait–Monotrait (HTMT) ratio, applying a conservative cutoff of 0.85.

##### 2.2.3.2 Validity Tests

For concurrent validity, correlations were examined between the newly developed self‐efficacy scale and the Korean version of the PSCNI (Song and Noh 1996). The PSCNI is a translated and validated version of Arthur (1990) original instrument and it is widely used; thus, it was deemed an appropriate criterion for evaluating concurrent validity in this study.

For convergent validity, correlations were assessed between the new scale and the Maslach Burnout Inventory–Human Services Survey for Medical Personnel (MBI‐HSS (MP)) (Maslach and Jackson 1981). PSC is a key determinant of nurses' burnout, with higher self‐concept associated with lower burnout (Kim and Jung 2022). Accordingly, a significant negative correlation with MBI‐HSS (MP), a widely used measure of burnout among healthcare professionals, was specified a priori and interpreted as evidence of convergent validity (Rönkkö and Cho 2022). Concurrent and convergent validity were tested using Pearson correlation coefficients between the new scale and each criterion measure.

##### 2.2.3.3 Reliability Testing

Reliability was evaluated using Cronbach's alpha and McDonald's ω. Cronbach's alpha is influenced by several statistical assumptions such as tau‐equivalence and continuous distribution, whereas McDonald's ω provides more stable reliability estimates by utilizing CFA‐based factor loadings and error variances. Therefore, following the recommendation for a multifaceted approach that reports both alpha and omega coefficients (Kalkbrenner 2024), we evaluated both Cronbach's alpha and McDonald's ω.

#### 2.2.4 Step 9: Scale Finalization

Based on the results of validity and reliability testing of the PSC scale for hospital nurses, the length of the instrument was optimized.

### 2.3 Data Analysis

The data were analyzed using the statistical programs SPSS/WIN 29.0 and R 4.5.1. The general characteristics of the participants were analyzed using descriptive statistics such as percentages, frequencies, means, and standard deviations. Data screening for missing values and outliers was performed in R. Content validity was assessed with the item content validity index (I‐CVI) and scale‐level content validity index, averaging method (S‐CVI/Ave). EFA and CFA, as well as validity and reliability testing, were conducted in R.

### 2.4 Ethical Consideration

This study was conducted after obtaining approval from the Institutional Review Board of The Catholic University of Korea (IRB No. MC22EASI0129). All participants were informed in detail about the objectives and purpose of the study, procedures and methods, time required to complete the survey, and collection of personal information. Only those who were willing to participate voluntarily were included.

## 3 Results

### 3.1 Scale Development

The literature review yielded a total of nine construct factors and 39 attributes, which include two construct factors for the social self‐concept domain (relationship and leadership), four for the emotional self‐concept domain (satisfaction, autonomy, psychological empowerment, and self‐efficacy), two for the academic self‐concept domain (knowledge and professionalism), and one for the physical self‐concept domain (environment).

After identifying the attributes of the PSC of hospital nurses derived from the literature review, focus group interviews were conducted to determine perceptions and conceptual factors related to PSC. The focus group interviews were conducted with seven nurses and resulted in the addition of the following seven attributes not identified in the literature review: satisfaction derived from rewards, a sense of achievement through continuous learning, professional values, willingness toward self‐development, recognition of professionalism through patients' trust, patient‐centered thinking, and the influence of social perception. The focus group interviews ultimately resulted in nine factors and 46 attributes.

A five‐point Likert scale was employed for scoring the scale. Based on the first round of content validation, one item with an I‐CVI of less than 0.80 and five items with overlapping meanings were excluded, while two items were added to the item pool. Based on the experts' comments, the items were modified and supplemented, which left a total of 49 preliminary items. In the second round of validation, two items with an I‐CVI of less than 0.80 were excluded. A total of three items were modified based on the opinions of experts. Finally, a total of 47 items that met the content validation criteria were selected. Next, these 47 items were assessed by a Korean language instructor for the flow of sentences, grammatical accuracy, and appropriateness of expressions, followed by modifications and syntax improvements as needed.

#### 3.1.1 Pilot Testing Results

The mean length of total clinical experience was 9.33 years, and the mean working duration in the current hospital was 8.93 years. Regarding education level, 12 participants had master's degrees or higher. The most common job position was staff nurse (*n* = 14), and the most common department was the internal medicine ward (*n* = 12). A total of 22 participants reported that their work schedule was three shifts, while seven nurses worked day shift. Participants responded that the readability and length of all the items were reasonable and ambiguity was minimal. The mean time spent answering the questionnaire was 6.93 min. The mean level of understanding for each item was 3.06 points. Regarding the ambiguity of the items, no responses of “No” were given. However, three items that might result in different answers depending on the respondent were modified, leading to the establishment of a final set of 47 preliminary items.

### 3.2 Scale Verification

#### 3.2.1 Main Survey Results

##### 3.2.1.1 General Characteristics of the Participants

Of the 440 participants, 396 participants were female, and the mean age was 32.56 years. Total clinical experience ranged from 12 to 444 months, and clinical experience in the current hospital ranged from 12 to 408 months. In terms of current position, 335 were staff nurses and 10 were nurse managers. In terms of work department, 149 were working in the internal medicine ward (Table 2).

**Table 2:** General characteristics of the study participants (*N* = 440).

Variable	Categories	Total (*N* = 440)		EFA (*n* = 220)		CFA (*n* = 220)		χ^2^ or t	ρ
		*n*	%	*n*	%	or M	SD		
Gender	Male	44	10.00	25	11.36	19	8.64	0.909	0.340
	Female	396	90.00	195	88.64	201	91.36		
Age (years)^a^	Range (23–58)	32.56	5.94	32.22	5.84	32.90	6.03	1.205	0.229
Marital status	Married	156	35.45	79	35.91	77	35.00	1.029	0.598
	Single	283	64.32	141	64.09	142	64.55		
	Other	1	0.23	0	0	1	0.45		
Education level	Associate's degree	58	13.18	23	10.45	35	15.91	2.969	0.227
	Bachelor's degree	296	67.27	154	70.00	142	64.55		
	Master's degree or above	86	19.55	43	19.55	43	19.55		
Religion	Yes	183	41.59	84	38.18	99	45.00	2.105	0.147
	No	257	58.41	136	61.82	121	55.00		
Total clinical experience^a^ (months)	Range (12–444)	101.77	68.84	97.42	66.61	106.12	70.88	1.327	0.185
Current hospital experience^a^ (months)	Range (12–408)	86.10	69.68	84.78	68.05	87.41	71.41	0.396	0.692
Current position	Staff nurse	335	76.14	174	79.09	161	73.18	2.178	0.337
	Charge nurse	95	21.59	42	19.09	53	24.09		
	Nurse manager	10	2.27	4	1.82	6	2.73		
Department	Internal medicine ward	149	33.86	74	33.64	75	34.09	1.945	0.746
	Surgical ward	124	28.16	60	27.27	64	29.09		
	Emergency room	22	5.00	14	6.36	8	3.64		
	Specialized department	89	20.23	43	19.55	46	20.91		
	Outpatient and clinic center	56	12.73	29	13.18	27	12.27		
Work schedule	2 shifts	21	4.77	10	4.55	11	5.00	0.058	0.971
	3 shifts	322	73.18	161	73.18	161	73.18		
	Day shift	97	22.05	49	22.27	48	21.82		

Abbreviations: M, mean; SD, standard deviation.

^a^ M (SD), independent samples *t*‐test.

### 3.3 Analysis of Items

The item‐total correlation indicated that no items had an item‐total correlation coefficient of less than 0.30 (Table 3).

**Table 3:** Item analysis results (*N* = 440).

Items	M	SD	Skew.	Kurt.	CITC	α
1.	3.78	0.86	−0.405	−0.199	0.646	0.963
2.	4.08	0.59	−0.153	0.333	0.546	0.964
3.	3.93	0.67	−0.412	0.488	0.604	0.964
4.	3.94	0.73	−0.794	1.455	0.493	0.964
5.	4.20	0.69	−0.693	0.991	0.510	0.964
6.	4.06	0.70	−0.552	0.532	0.491	0.964
7.	4.01	0.79	−0.692	0.800	0.549	0.964
8.	3.57	0.98	−0.500	−0.040	0.668	0.963
9.	3.95	0.73	−0.469	0.221	0.599	0.964
10.	3.56	0.96	−0.374	−0.540	0.674	0.963
11.	3.39	1.03	−0.353	−0.472	0.637	0.963
12.	3.38	1.07	−0.192	−0.816	0.604	0.964
13.	3.58	0.82	−0.318	−0.059	0.631	0.964
14.	3.09	1.15	−0.034	−0.916	0.633	0.964
15.	3.62	0.93	−0.630	0.110	0.701	0.963
16.	3.48	1.07	−0.458	−0.319	0.693	0.963
17.	4.08	0.66	−0.282	−0.037	0.591	0.964
18.	3.96	0.76	−0.543	0.206	0.572	0.964
19.	3.90	0.77	−0.554	0.566	0.680	0.963
20.	4.34	0.60	−0.498	0.435	0.447	0.964
21.	3.75	1.01	−0.845	0.319	0.565	0.964
22.	3.71	1.03	−0.722	0.030	0.649	0.963
23.	4.08	0.89	−0.997	0.871	0.519	0.964
24.	3.95	0.90	−0.770	0.439	0.634	0.963
25.	4.14	0.71	−0.929	2.485	0.569	0.964
26.	3.89	0.95	−0.803	0.536	0.630	0.963
27.	3.95	0.82	−0.819	1.092	0.575	0.964
28.	3.67	0.96	−0.591	−0.185	0.599	0.964
29.	3.77	0.87	−0.634	0.358	0.614	0.964
30.	4.00	0.83	−0.834	1.044	0.606	0.964
31.	2.69	1.27	0.288	−0.978	0.669	0.963
32.	2.91	1.13	0.097	−0.750	0.624	0.964
33.	3.99	0.75	−0.706	1.066	0.558	0.964
34.	3.84	0.75	−0.469	0.502	0.595	0.964
35.	4.10	0.64	−0.405	0.949	0.522	0.964
36.	4.03	0.63	−0.075	−0.240	0.637	0.964
37.	4.08	0.64	−0.172	−0.196	0.568	0.964
38.	4.08	0.62	−0.283	0.420	0.576	0.964
39.	4.06	0.64	−0.365	0.535	0.615	0.964
40.	3.89	0.80	−0.754	0.911	0.647	0.963
41.	3.69	0.91	−0.670	0.381	0.668	0.963
42.	3.27	1.15	−0.206	−0.854	0.564	0.964
43.	3.07	1.17	−0.013	−0.897	0.628	0.964
44.	2.86	1.17	0.178	−0.812	0.584	0.964
45.	2.65	1.19	0.302	−0.835	0.653	0.963
46.	2.86	1.20	0.177	−0.884	0.688	0.963
47.	2.96	1.18	−0.059	−0.929	0.585	0.964

Abbreviations: M, mean; SD, standard deviation; Skew., skewness; Kurt., kurtosis; CITC, corrected item‐total correlation; α, Cronbach's alpha.

#### 3.3.1 Verification of Construct Validity

Prior to conducting factor analyses, missing values and outliers were examined. No missing data (0%) or univariate outliers (|*z* **|** > 3.29) were identified. However, 24 multivariate outliers were detected based on Mahalanobis distance (χ² [47] = 82.72, *p* **<** 0.001). Of these, 13 were identified in the EFA sample and 11 in the CFA sample. These cases were not immediately removed; instead, the final 23‐item model was established using the full sample, and sensitivity analyses were subsequently conducted to compare the results with and without the identified outliers.

##### 3.3.1.1 EPA

The suitability of the data for EFA was confirmed by the KMO test and Bartlett's test of sphericity. Initially, EFA was performed on 47 items. To determine the optimal number of factors, both parallel analysis and the scree plot were examined, resulting in a five‐factor solution. In the first EFA, 18 items were removed from the original 47 items, including 10 items with factor loadings below 0.50, three items with cross‐loadings, and five items with communalities below 0.50. A second EFA was then conducted with the remaining 29 items, which resulted in the removal of four items (two with cross‐loadings and two with communalities below 0.50). A third EFA was performed with 25 items, after which two items with factor loadings below 0.50 were eliminated. Finally, factor analysis was conducted with 23 items. The KMO value was 0.871, indicating excellent sampling adequacy. Bartlett's test of sphericity was significant (*p* < 0.001), supporting the suitability for EFA. Communalities ranged from 0.508 to 0.874, and the cumulative explained variance was 65.00%. (Table 4).

**Table 4:** Exploratory factor analysis results (*n* = 220).

Items	Communality	F1	F2	F3	F4	F5
17. I am a helpful nurse for patients.	0.628	0.704	−0.031	0.186	−0.015	−0.012
34. I strive to improve my nursing practice for my patients.	0.508	0.582	0.001	0.101	0.152	−0.024
35. I strive to provide quality nursing care to my patients.	0.634	0.824	−0.146	0.152	0.026	−0.106
37. I can educate patients or their caregivers using my nursing knowledge.	0.658	0.823	0.095	−0.042	−0.156	0.010
38. I can apply what I have learned to my nursing work.	0.664	0.799	0.009	−0.014	−0.080	0.087
39. I have expertise in nursing.	0.614	0.713	0.075	−0.093	0.125	0.029
40. I can teach new knowledge about nursing practice to my colleagues.	0.662	0.715	0.201	−0.174	0.075	0.037
10. I feel that I can handle any nursing duties.	0.710	−0.011	0.784	0.070	0.068	−0.027
11. I am confident that I can handle any new nursing duties (changes in department, job responsibilities, roles, etc.).	0.594	−0.046	0.780	0.060	−0.040	0.024
12. I am not afraid to apply new medical technologies (medical equipment, new drugs, etc.) to my work.	0.605	0.025	0.779	−0.030	−0.066	0.068
13. I can deal with unexpected situations with competence when performing nursing duties.	0.599	0.179	0.678	−0.027	−0.080	0.065
14. I am not afraid of emergencies that occur when performing nursing duties.	0.715	0.030	0.851	0.018	0.053	−0.149
16. I enjoy working as a nurse.	0.666	−0.011	0.179	0.794	−0.030	−0.090
21. I think the nursing profession can continue to evolve.	0.575	−0.092	−0.008	0.657	0.149	0.100
22. I will continue to work as a nurse.	0.645	−0.054	0.135	0.721	0.069	−0.004
23. I think nursing is a valuable profession.	0.577	0.108	−0.089	0.785	−0.152	0.042
24. I feel a sense of mission as a nurse.	0.682	0.105	−0.074	0.751	0.038	0.039
42. I think that nursing is a socially recognized profession.	0.753	0.093	−0.127	−0.012	0.927	−0.042
43. I think nursing is recognized as a professional occupation.	0.874	−0.024	−0.047	−0.002	0.906	0.130
44. I think my nursing work is legally protected.	0.610	−0.093	0.266	0.023	0.652	−0.059
4. I feel that I cooperate well withmy colleagues.	0.696	0.048	−0.075	0.091	−0.045	0.810
5. I have a respectful relationship with my colleagues.	0.685	−0.007	−0.052	0.007	0.002	0.850
7. I enjoy working with my colleagues.	0.597	−0.023	0.169	−0.018	0.089	0.648
Parallel analysis eigenvalue (95th percentile)		1.096	0.936	0.851	0.799	0.714
Observed eigenvalue		10.165	2.121	1.997	1.304	1.041
Explained variance (%)		18.06	14.93	13.39	10.07	8.55
Total explained variance (%)		18.06	32.99	46.38	56.45	65.00

*Note:* F1, Nursing Job Competency; F2, Nursing Job Self‐Efficacy; F3, Nursing Job Identity; F4, Nursing Professional Perception; F5, Nursing Collegial Relationship Satisfaction.

Factor 1 consisted of seven items assessing professional nursing knowledge and competence, the ability to apply professional knowledge, and willingness toward continuous improvement (Nursing Job Competency). Factor 2 consisted of five items measuring nurses' confidence in job performance (Nursing Job Self‐Efficacy). Factor 3 consisted of five items assessing nurses' positive perception of their work and sense of mission (Nursing Job Identity). Factor 4 consisted of three items measuring the degree of recognition of nurses' professionalism (Nursing Professional Perception). Factor 5 consisted of three items measuring cooperation with colleagues and satisfaction with workplace relationships (Nursing Colleagial Relationship Satisfaction).

To examine robustness, sensitivity analysis was conducted by excluding multivariate outliers identified using Mahalanobis distance (χ² cutoff = 49.73, df = 23, α = 0.001). Nine cases (4.09%) were removed, reducing the sample to *n* = 211. The resulting factor solution showed a slightly higher total explained variance (65.78%) but a somewhat lower KMO (0.848). Bartlett's test remained significant (χ²(253) = 3751.42, *p* < 0.001). As the factor structure and reliability were essentially unchanged, the results from the full sample were reported.

##### 3.3.1.2 CFA

**Figure 2:**
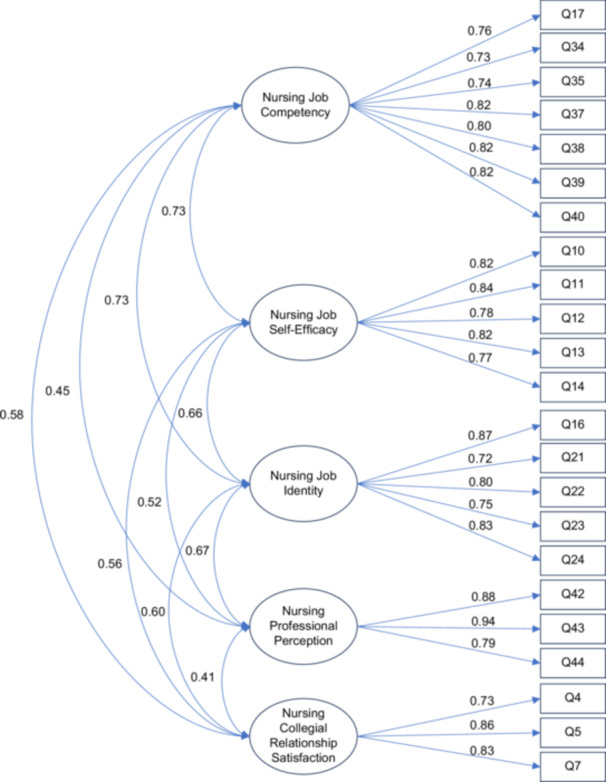
Final confirmatory factor analysis model results.

CFA was conducted to evaluate the model fit of the five‐factor, 23‐item structure using data from 220 participants. The results indicated a good model fit: χ²(220) = 307.238, *p* < 0.001, χ²/df = 1.397, GFI = 0.989, CFI = 0.996, TLI = 0.995, SRMR = 0.058 and RMSEA = 0.043 (90% CI = 0.031–0.053). Standardized factor loadings ranged from 0.723 to 0.939, all of which exceeded the recommended threshold of 0.50, supporting convergent validity among factors. (Figure 2; Table 5).

**Table 5:** Factor convergent validity of the PSCS‐HN (*n* = 220).

Factor	Item	Standardized estimates	SE	*z*	AVE	Construct reliability
Nursing job competency	Q17	0.756			0.617	0.918
	Q34	0.733	0.079	12.261		
	Q35	0.741	0.068	14.462		
	Q37	0.816	0.066	16.243		
	Q38	0.801	0.081	13.018		
	Q39	0.823	0.068	15.971		
	Q40	0.821	0.072	15.169		
Nursing job self‐efficacy	Q10	0.817			0.651	0.903
	Q11	0.842	0.061	16.952		
	Q12	0.781	0.066	14.470		
	Q13	0.816	0.060	16.560		
	Q14	0.775	0.058	16.325		
Nursing job identity	Q16	0.866			0.634	0.896
	Q21	0.723	0.055	15.108		
	Q22	0.802	0.052	17.678		
	Q23	0.749	0.055	15.677		
	Q24	0.833	0.050	19.366		
Nursing professional perception	Q42	0.882			0.763	0.906
	Q43	0.939	0.046	23.129		
	Q44	0.793	0.057	15.756		
Nursing collegial relationship satisfaction	Q4	0.734			0.655	0.850
	Q5	0.859	0.112	10.455		
	Q7	0.829	0.111	10.150		

*Note:* Standardized factor loadings are presented. Standard errors (SE) and *z* values are based on unstandardized parameter estimates. The first item of each factor was fixed to 1.0 for model identification. [Correction added on 3 March 2026, after first online publication: In Table 5, the SE value for ‘Nursing job competency’, Item Q34, has been corrected from “0.07” to “0.079” in this version.]

CFA with and without 11 multivariate outliers showed that model fit improved slightly after outlier removal (RMSEA = 0.043 → 0.032, CFI = 0.996 → 0.998), while the factor structure remained unchanged. Standardized loadings were stable (0.723–0.939 before vs. 0.710–0.926 after, all > 0.70), supporting convergent validity. Reliability (CR = 0.85–0.92; AVE = 0.62–0.76) was adequate. Therefore, the final model was confirmed based on the full sample without excluding the outliers.

Discriminant factor validity was confirmed by both the Fornell–Larcker criterion and HTMT. For all factors, the AVE values (0.617–0.763) exceeded the highest squared correlations with other constructs (0.170–0.537). In addition, the highest HTMT estimate was below 0.80, well under the conservative threshold of 0.85. These results demonstrate that discriminant validity was adequately established.

##### 3.3.1.3 Verification of Concurrent Validity

Significant positive correlations were found with the PSCS‐HN and PSCNI, with factor‐level correlation coefficients ranging from *r* = 0.508 to *r* = 0.700 (*p* < 0.001), and an overall scale correlation coefficient of *r* = 0.793 (*p* < 0.001).

##### 3.3.1.4 Verification of Convergent Validity

Significant negative correlations were found with the PSCS‐HN and MBI‐HSS (MP), with factor‐level correlation coefficients ranging from *r* = –0.461 to *r* = –0.603 (*p* < 0.001), and an overall scale correlation coefficient of *r* = –0.715 (*p* < 0.001). Although the correlation between the PSCS‐HN and the MBI‐HSS(MP) was relatively high, it did not approach the level (*r* ≥ 0.85) typically considered indicative of construct redundancy (Cheung et al. 2024). Indicating that professional self‐concept and burnout are related yet conceptually distinct constructs.

#### 3.3.2 Reliability Testing

Internal consistency reliability was examined using Cronbach's alpha and McDonald's ω. Cronbach's alpha values were 0.864 for F1, 0.869 for F2, 0.858 for F3, 0.859 for F4, and 0.794 for F5, with an overall reliability of 0.927 for the 23‐item scale. McDonald's ω values showed a similar pattern (ω = 0.796–0.873 across factors; total scale ω = 0.953), thus confirming the reliability of the PSCS‐HN.

## 4 Discussion

In this study, we developed the PSCS‐HN based on DeVellis and Thorpe's (2021) scale development guidelines and verified its validity and reliability. In the scale development stage, 53 preliminary items were developed based on a literature review and focus group interviews, after which content validity was verified by two rounds of expert panel reviews, resulting in a refined set of 47 preliminary items. In the scale evaluation stage, a survey was conducted with 440 nurses to verify the validity and reliability of the scale. Evaluation of construct validity through item analysis, EFA, and CFA resulted in a total of 23 items with five factors. The explanatory power of the scale was 65.00%. Each item was scored on a five‐point Likert scale. The range of scores was 23–115, with a higher score indicating a higher PSC.

Factor 1, “Nursing Job Competency,” consisted of seven items on professional nursing knowledge and application, teaching ability, and clinical application ability. Hospital nurses work on the front lines, providing patient care and performing professional duties, thus requiring them to maintain continuing competence. The related duties help nurses integrate knowledge, skills, attitudes, and critical thinking abilities to perform their tasks effectively (Mrayyan et al. 2023). This factor is consistent with previous research indicating that among the domains of nursing competency, professional practice, which includes leadership in guiding others and skills to perform one's work skillfully, has the greatest influence (Seo et al. 2017). Therefore, competency on nursing job tasks should be cultivated to achieve hospital nurses' PSC.

Factor 2, “Nursing Job Self‐Efficacy,” consisted of five items regarding confidence in job performance, emotional stability, and self‐confidence in nursing duties. These defining attributes of job‐related self‐efficacy are similar to the findings of previous research indicating that self‐efficacy is an important factor in nurses' job performance, which motivates them to continuously learn and grow and contributes to further improvements in their expertise (Yao et al. 2021). The item “I am not afraid to apply new medical technologies (medical equipment, new drugs, etc.) to my work” aligns with previous findings; nurses feel the need for proficient skills appropriate to match advancing medical technologies, and the introduction of medical equipment enhances the professional image of nurses, thus indicating a positive correlation between perceptions of developments in medical technology and PSC (Barchielli et al. 2021). As self‐efficacy may play a role in helping nurses acquire new knowledge and skills, it should be a crucial component of hospital nurses' PSC.

Factor 3, “Nursing Job Identity,” was composed of five items, including a sense of mission as a professional, job satisfaction, and job advancement expectations. Professional identity refers to the self‐image of a nurse performing a professional role in work‐related circumstances or environments. Nurses are expected to establish an identity as nursing professionals to clearly understand their roles and responsibilities and also improve the quality of their nursing care for patients (Pizziconi et al. 2021). The item “I think the nursing professional can continue to evolve” is consistent with the results of previous research suggesting that continuous professional development positively affects one's professional identity (Philippa et al. 2021). The fact that hospital nurses' PSC can be developed when their job identity as a nurse is firmly formed should be especially noted.

Factor 4, “Nursing Professional Perception,” consisted of items addressing nurses' social status, recognition of their professionalism, and institutional protection. This factor includes items derived from statements about experienced nurses' recognition as professionals and their legal protection—elements that had not been presented in previously developed instruments. The legal status and protection of nurses are critical factors enabling Korean nurses to perform their duties safely. For example, during the COVID‐19 pandemic, the role of nurses was emphasized and the importance of their legal status and protection was highlighted (Wilson et al. 2020). An environment in which nurses are socially recognized and legally protected has significant implications for strengthening PSC. This aligns with previous findings that PSC serves a mediating role in the relationship between sociocultural factors and public image (Sacgaca et al. 2024). Therefore, to enhance PSC, it is essential to establish a work environment in which nurses receive both social recognition and legal protection.

Factor 5, “Nursing Collegial Relationship Satisfaction,” consisted of three items related to collegial cooperation, satisfaction with collegial relationships, and mutual respect. These items provide an indication of the level of a nurse's PSC based on the relationship with colleagues. This result is consistent with previous findings suggesting that PSC increases when nurses are respected by their colleagues (Parandavar et al. 2015). As hospital nurses' PSC is related to their relational satisfaction with nursing colleagues, increasing support from colleagues can be an important strategy to enhance hospital nurses' PSC.

CFA results confirm convergent and discriminant validity of the five factors, thereby indicating that each factor is composed of items that have not only common attributes but also conceptual distinction between one another. With a total of 23 items displaying an internal consistency reliability of 0.927, the PSCS‐HN is suitable for measuring hospital nurses' PSC. The PSCS‐HN developed in this study differs from existing scales in that it reflects the cultural, social, and work environment of hospital nurses in a comprehensive way and was designed exclusively for such nurses.

Regarding the significance of this study from the perspective of nursing practice, the PSCS‐HN can be used as a fundamental resource for developing strategies for efficient nursing workforce management as it allows assessment of hospital nurses' PSC. Such data are expected to help improve the quality of nursing care by increasing nurses' job satisfaction and reducing turnover.

Regarding the significance of this study for nursing health services research, the attributes of PSC identified could be widely employed in diverse studies on hospital nurses' PSC. Hospital nurses can evaluate their PSC using the scale and recognize their shortcomings. This finding may serve as a key indicator for the management and evaluation of organizational commitment and the quality of nursing services. Moreover, it can be used as an analytical tool to examine variations in PSC among hospital nurses across different hospital types and regions, thereby providing robust empirical evidence to inform the development of nursing health service policies.

The significance of this study in nursing education lies in the fact that, based on the measurement results of this research tool, nurses can examine their PSC, recognize areas needing improvement, and thereby gain opportunities for development. By objectively assessing nurses' levels of PSC, the findings can be utilized to design educational programs that strengthen nurses' growth plans and professional competencies, ultimately contributing to the enhancement of nursing quality.

### 4.1 Limitations

This study has some limitations that should be acknowledged. First, only nurses from departments at a general hospital or higher‐level healthcare institution and those whose work involved direct interactions with patients and their guardians were included, which limits the generalizability of the results. Second, the reliability of the scale was verified through an anonymous online survey of nurses nationwide, which limits the test‐retest reliability of the scale. Third, the focus group interviews participants were nurses working in Seoul, thus limiting the diversity of responses. The adequacy of sample size for EFA and CFA should be evaluated in relation to key characteristics of the tested model, such as model complexity, indicator quality, and overall model fit, rather than by a single numerical criterion. Although the present study considered these factors and demonstrated strong factor loadings and good model fit, replication with larger and more diverse samples is warranted to further confirm the robustness and generalizability of the findings. Despite these limitations, this study can be considered significant for its development of a reliable and valid scale that can be applied to measure hospital nurses' PSC.

## 5 Conclusion

The PSCS‐HN, which comprises 23 items and five factors, is a scale used to measure hospital nurses' PSC. The validity and reliability of the scale were verified. Further research should be conducted whith a larger and more diverse samples to generalize the study findings and expand the applicability of the scale.

## Author Contributions

**Eun‐Ha Kim:** Conceptualization, methodology, investigation, formal analysis, writing – original draft preparation, writing – review and editing. **Hye‐Ah Yeom:** conceptualization, methodology, supervision, writing – review and editing.

## Funding

The authors received no specific funding for this work.

### 1 Conflicts of Interest

The authors declare no conflicts of interest.

## Data Availability

The data that support the findings of this study are not publicly available due to ethical restrictions and participant privacy. In accordance with the Institutional Review Board (IRB)–approved protocol, survey data are retained only for purposes permitted by the IRB and will be destroyed after completion of all research and publication procedures, while focus group interview data are stored for 3 years and will be destroyed thereafter.

## 1

Table A1

**Table A1:** Final items.

Items	Strongly disagree	Disagree	Neutral	Agree	Strongly agree
1. I am a helpful nurse for patients.					
2. I strive to improve my nursing practice for my patients.					
3. I strive to provide quality nursing care to my patients.					
4. I can educate patients or their caregivers using my nursing knowledge.					
5. I can apply what I have learned to my nursing work.					
6. I have expertise in nursing.					
7. I can teach new knowledge about nursing practice to my colleagues.					
8. I feel that I can handle any nursing duties.					
9. I am confident that I can handle any new nursing duties (changes in department, job responsibilities, roles, etc.).					
10. I am not afraid to apply new medical technologies (medical equipment, new drugs, etc.) to my work.					
11. I can deal with unexpected situations with competence when performing nursing duties.					
12. I am not afraid of emergencies that occur when performing nursing duties.					
13. I enjoy working as a nurse.					
14. I think the nursing profession can continue to evolve.					
15. I will continue to work as a nurse.					
16. I think nursing is a valuable profession.					
17. I feel a sense of mission as a nurse.					
18. I think that nursing is a socially recognized profession.					
19. I think nursing is recognized as a professional occupation.					
20. I think my nursing work is legally protected.					
21. I feel that I cooperate well with my colleagues.					
22. I have a respectful relationship with my colleagues.					
23. I enjoy working with my colleagues.					

## References

[nur70059-bib-0001] Angel, E. , R. Craven , and N. Denson . 2012. “The Nurses Self‐Concept Instrument (NSCI): Assessment of Psychometric Properties for Australian Domestic and International Student Nurses.” International Journal of Nursing Studies 49, no. 7: 880–886. 10.1016/j.ijnurstu.2012.01.016. 22356798

[nur70059-bib-0002] Arthur, D. 1992. “Measuring the Professional Self‐Concept of Nurses: A Critical Review.” Journal of Advanced Nursing 17, no. 6: 712–719. 10.1111/j.1365-2648.1992.tb01969.x. 1607504

[nur70059-bib-0003] Arthur, D. 1995. “Measurement of the Professional Self‐Concept of Nurses: Developing a Measurement Instrument.” Nurse Education Today 15, no. 5: 328–335. 10.1016/S0260-6917(95)80004-2. 7494526

[nur70059-bib-0004] Arthur, D. G. (1990). *The development of an instrument for measuring the professional self‐concept of nurses* [Doctoral dissertation, University of Newcastle].

[nur70059-bib-0005] Barchielli, C. , C. Marullo , M. Bonciani , and M. Vainieri . 2021. “Nurses and the Acceptance of Innovations in Technology‐Intensive Contexts: The Need for Tailored Management Strategies.” BMC Health Services Research 21: 639. 10.1186/s12913-021-06628-5. 34215228 PMC8253682

[nur70059-bib-0006] Cheung, G. W. , H. D. Cooper‐Thomas , R. S. Lau , and L. C. Wang . 2024. “Reporting Reliability, Convergent and Discriminant Validity with Structural Equation Modeling: A Review and Best‐Practice Recommendations.” Asia Pacific Journal of Management 41, no. 2: 745–783. 10.1007/s10490-023-09871-y.

[nur70059-bib-0008] Cowin, L. 2001. “Measuring Nurses' Self‐Concept.” Western Journal of Nursing Research 23, no. 3: 313–325. 10.1177/01939450122045177. 11291434

[nur70059-bib-0009] DeVellis, R. F. , and C. T. Thorpe . 2021. Scale development: Theory and applications (5th ed. SAGE Publications.

[nur70059-bib-0010] Hinkin, T. R. 1998. “A Brief Tutorial on the Development of Measures for Use in Survey Questionnaires.” Organizational Research Methods 1, no. 1: 104–121. 10.1177/109442819800100106.

[nur70059-bib-0011] Kalkbrenner, M. T. 2024. “Choosing Between Cronbach's Coefficient Alpha, Mcdonald's Coefficient Omega, and Coefficient H: Confidence Intervals and the Advantages and Drawbacks of Interpretive Guidelines.” Measurement and Evaluation in Counseling and Development 57, no. 2: 93–105. 10.1080/07481756.2023.2283637.

[nur70059-bib-0012] Kang, H. 2013. “A Guide on the Use of Factor Analysis in the Assessment of Construct Validity.” Journal of Korean Academy of Nursing 43, no. 5: 587–594. https://www.riss.kr/link?id=A99799008. 24351990 10.4040/jkan.2013.43.5.587

[nur70059-bib-0013] Kim, S. J. , and K. I. Jung . 2022. “The Influences of Professional Self‐Concept, Job Stress, and Coworker Support on Burnout in Oncology Unit Nurses.” Asian Oncology Nursing 22, no. 2: 104–110. 10.5388/aon.2022.22.2.104.

[nur70059-bib-0014] Kiwanuka, F. , J. Kopra , N. Sak‐Dankosky , R. C. Nanyonga , and T. Kvist . 2022. “Polychoric Correlation with Ordinal Data in Nursing Research.” Nursing Research 71, no. 6: 469–476. 10.1097/nnr.0000000000000614. 35997708 PMC9617753

[nur70059-bib-0015] Lynn, M. R. 1986. “Determination and Quantification of Content Validity.” Nursing Research 35, no. 6: 382–386. 10.1097/00006199-198611000-00017. 3640358

[nur70059-bib-0016] Maslach, C. 2003. Burnout: The Cost of Caring. ISHK.

[nur70059-bib-0017] Maslach, C. , and S. E. Jackson . 1981. “The Measurement of Experienced Burnout.” Journal of Organizational Behavior 2, no. 2: 99–113. 10.1002/job.4030020205.

[nur70059-bib-0018] Miao, C. , C. Liu , and Y. Zhou , et al. 2024. “Nurses' Perspectives on Professional Self‐Concept and Its Influencing Factors: A Qualitative Study.” BMC Nursing 23: 237. 10.1186/s12912-024-01834-y. 38594667 PMC11003037

[nur70059-bib-0019] Mrayyan, M. T. , H. Y. Abunab , and A. Abu Khait , et al. 2023. “Competency in Nursing Practice: A Concept Analysis.” BMJ Open 13, no. 6: e067352. 10.1136/bmjopen-2022-067352. PMC10255110 37263688

[nur70059-bib-0020] Parandavar, N. , A. Rahmanian , and Z. Badiyepeymaie Jahromi . 2015. “A Study of the Relationship between Nurses' Professional Self‐Concept and Professional Ethics in Hospitals Affiliated to Jahrom University of Medical Sciences, Iran.” Global Journal of Health Science 8, no. 4: 82–88. 10.5539/gjhs.v8n4p82. 26573035 PMC4873582

[nur70059-bib-0021] Park, E. J. , J. Y. Han , and N. Y. Jo . 2016. “Effects of Professional Self‐Concept, Self Efficacy on the Job Satisfaction in General Hospital Nurses.” Journal of the Korean Data and Information Science Society 27, no. 1: 191–201. http://www.riss.kr/link?id=A104378448.

[nur70059-bib-0022] Philippa, R. , H. Ann , M. Jacqueline , and A. Nicola . 2021. “Professional Identity in Nursing: A Mixed Method Research Study.” Nurse Education in Practice 52: 103039. 10.1016/j.nepr.2021.103039. 33823376

[nur70059-bib-0023] Pizziconi, V. , G. D'elpidio , C. Calandrella , and A. Gazzelloni . 2021. “Nursing Professional Identity: A Cross‐Sectional Study.” Professioni Infermieristiche 74, no. 4: 269. 10.7429/pi.2021.744269b. 35363992

[nur70059-bib-0024] Polit, D. F. , and C. T. Beck . 2006. “The Content Validity Index: Are You Sure You Know What's Being Reported? Critique and Recommendations.” Research in Nursing & Health 29, no. 5: 489–497. 10.1002/nur.20147. 16977646

[nur70059-bib-0025] Rönkkö, M. , and E. Cho . 2022. “An Updated Guideline for Assessing Discriminant Validity.” Organizational Research Methods 25, no. 1: 6–14. 10.1177/1094428120968614.

[nur70059-bib-0026] Sacgaca, L. , E. Pasayan , A. S. Alqarni , et al. 2024. “Sociocultural and Perceived Public Image of Nurses Among Nursing Students: The Mediating Role of Self‐Concept.” BMC Nursing 23, no. 1: 298. 10.1186/s12912-024-01957-2. 38689285 PMC11059634

[nur70059-bib-0027] Seo, M. S. , J. S. Park , O. K. Kim , Y. J. Lee , and H. J. Kim . 2017. “The Influence of Clinical Nurses' Professional Self‐Concept and Interpersonal Relations on Nursing Competence.” Journal of Hospital Management 22, no. 2: 28–43. http://www.riss.kr/link?id=A103227504.

[nur70059-bib-0028] Shavelson, R. J. , J. J. Hubner , and G. C. Stanton . 1976. “Self‐Concept: Validation of Construct Interpretations.” Review of educational research 46, no. 3: 407–441. 10.3102/00346543046003407.

[nur70059-bib-0029] Song, K. A. , and C. H. Noh . 1996. “An Analytical Study of the Professional Self‐Concept of Hospital Nurses in Korea.” Journal of Korean Academy of Nursing 26, no. 1: 94–106. http://www.riss.kr/link?id=A100114513.

[nur70059-bib-0030] Weis, D. , and M. J. Schank . 2002. “Professional Values: Key to Professional Development.” Journal of Professional Nursing 18, no. 5: 271–275. 10.1053/jpnu.2002.129224. 12434320

[nur70059-bib-0031] Wilson, R. L. , J. Carryer , and J. Dewing , et al. 2020. “The State of the Nursing Profession in the International Year of the Nurse and Midwife 2020 During COVID‐19: A Nursing Standpoint.” Nursing Philosophy 21, no. 3: e12314. 10.1111/nup.12314. 32706508 PMC7404428

[nur70059-bib-0032] Yao, X. , L. Yu , Y. Shen , Z. Kang , and X. Wang . 2021. “The Role of Self‐Efficacy in Mediating Between Professional Identity and Self‐Reported Competence among Nursing Students in the Internship Period: A Quantitative Study.” Nurse education in practice 57: 103252. 10.1016/j.nepr.2021.103252. 34781196

[nur70059-bib-0033] Zhou, L. , K. Sukpasjaroen , E. Cai , K. Moonsri , P. Imsiri , and T. Chankoson . 2023. “The Psychometric Properties of Nursing Image Measurement Instruments: A Systematic Review.” Nursing Open 10, no. 8: 5056–5078. 10.1002/nop2.1742. 37086148 PMC10333846

